# Persistent Zika Virus Detection in Semen in a Traveler Returning to the United Kingdom from Brazil, 2016 

**DOI:** 10.3201/eid2301.161300

**Published:** 2017-01

**Authors:** Katherine M. Gaskell, Catherine Houlihan, Eleni Nastouli, Anna M. Checkley

**Affiliations:** Hospital for Tropical Diseases at University College London Hospital, London, UK (K.M. Gaskell, A.M. Checkley);; London School of Hygiene and Tropical Medicine, London (K.M. Gaskell);; University College London Hospitals Department of Clinical Virology, London (C. Houlihan, E. Nastouli);; Department of Population, Policy and Practice, University College London Great Ormond Street Institute of Child Health, London (E. Nastouli)

**Keywords:** Zika virus, RNA detection, isolation, asymptomatic, infection, infectious diseases, semen, persistence, sexual transmission risk, viruses, vector-borne infections

## Abstract

Zika virus is normally transmitted by mosquitos, but cases of sexual transmission have been reported. We describe a patient with symptomatic Zika virus infection in whom the virus was detected in semen for 92 days. Our findings support recommendations for 6 months of barrier contraceptive use after symptomatic Zika virus infection.

Zika virus is a mosquito-transmitted flavivirus that was first isolated from mosquitos in the Zika Forest in Uganda. Since its introduction into Brazil in May 2015, the virus has spread rapidly through the Americas, and transmission is now widespread in South America, Central America, the Caribbean, the Pacific islands, Singapore, and Thailand (*1*). The virus is transmitted mainly by *Aedes aegypti* mosquitos and causes symptoms in ≈20% of persons infected (*2*), usually manifesting as a mild illness consisting of fever, arthralgia, myalgia, conjunctivitis, and pruritic rash. Infection in pregnancy can lead to congenital Zika syndrome, which consists of multiple developmental abnormalities, including microcephaly and cerebral calcification, and fetal loss. Zika virus infection is also associated with Guillain-Barré syndrome (*3*).

Although by far the most common route of Zika virus transmission is by mosquito, the virus can also be transmitted sexually (from male to female, female to male, and male to male) (*4*–*8*). Zika virus RNA has been detected in semen and vaginal fluid (*9*–*12*). We describe a case in which Zika virus persisted in semen for 92 days after symptom onset.

## The Study 

A previously healthy 45-year-old man became ill 1 day after returning to the United Kingdom from a 1-week holiday in Rio de Janeiro, Brazil, in February 2016. His illness lasted for 10 days and consisted of severe retro-orbital headache, arthralgia, myalgia, and high fevers, followed by a pruritic maculopapular rash. He had no symptoms of prostatitis or gross hematospermia. He had no history of immunocompromise, and he was not on any regular medication. On day 3 of his illness, Zika virus RNA was detected in urine but not in serum; Zika virus IgM and IgG were not detected in serum by an ELISA IgM and IgG kit (EUROIMMUN AG, Lübeck, Germany) (*13*). Results of serologic testing for chikungunya, dengue, and yellow fever were negative by commercial assays. 

Seventeen days after the initial testing, Zika virus IgM and IgG were detected in a new serum sample from the patient. The patient and his partner were planning to conceive and were reluctant to wait the 6 months recommended by Public Health England (*1*). Therefore, PCR for Zika virus RNA was performed on serial semen samples by using the Rare and Imported Pathogens Laboratory’s in-house real-time reverse transcription PCR (rRT-PCR), based on an assay used by Pyke et al. (*14*). Zika virus RNA was detected 22, 55, and 92 days after symptom onset (cycle threshold values 21.3, 30.1, and 37.2, respectively). No microhematospermia was detected, and Zika virus could not be cultured at any point. No Zika virus RNA was detected in semen at day 132 or day 174. The patient and his partner did not have unprotected sex during this period; his partner remained well and was not tested for Zika virus.

## Conclusions

In this case, Zika virus RNA was detected in the semen of a previously healthy, immunocompetent adult who contracted his infection during a short visit to Rio de Janeiro, Brazil, in February 2016. RNA was present in semen samples until 92 days after the onset of symptoms and was subsequently undetectable on 2 occasions.

This report follows several others from the current outbreak in which Zika virus RNA has been detected in semen >90 days after symptom onset in an immunocompetent and previously healthy adult. Previous reports include a man who had visited Haiti, in whom Zika virus RNA was persistently identified in semen until day 188 after symptom onset (*10*). A subsequent report from Italy documented Zika virus RNA being persistently identified in semen until day 181 in a symptomatic traveler returning from Haiti (*11*). In these 2 cases, unlike the present case, disappearance from semen was not demonstrated at a later point in time. In another case, Zika virus RNA was detected in semen 93 days after symptom onset; in that case, the patient had traveled to a nonepidemic area and had just been diagnosed with a sarcoma, and treatment including aggressive chemotherapy was planned (*12*). This subsequent treatment might have altered the kinetics of virus clearance. Further clarification on whether both symptomatic and asymptomatic patients can persistently shed Zika virus RNA in semen for prolonged periods is needed.

This case is important because it reinforces the possibility raised by Nicastri et al. (*10*) that Zika virus might be sexually transmitted at later points in time than have been documented thus far. The longest known period between Zika virus infection and sexual transmission to another person is 31–42 days after onset of symptoms. It is unclear how the presence of Zika virus (or level of RNA) in semen correlates to infectivity, or how long a person might be infectious by this route after infection with Zika virus. Guidelines from the World Health Organization, Public Health England, and the Centers for Disease Control and Prevention all recommend avoiding unprotected sex for 6 months after symptomatic Zika virus infection in a man (*1*,*15*). Published literature on sexual transmission and detection of Zika virus RNA in semen, including our report, supports these guidelines. As of press time for this article, published recommendations differ on how long to avoid unprotected sex in asymptomatic couples who have been potentially exposed to the virus.

An important caveat, however, is that virus could not be cultured from semen samples from this patient or from the patients reported by Nicastri et al. (*10*) or Barzon et al. (*11*) at any point. This findings raise the possibility that the detection of RNA does not equate to the detection of infectious virus particles.

An inability to detect Zika virus RNA in semen has been assumed to equate to a lack of infectivity by the sexual route (*10*,*11*). In this case, in which our patient and his partner planned to try to conceive, the results were useful in encouraging them to defer their plans until 2 sequential specimens were negative for Zika virus RNA. This delay, in fact, corresponded to 6 months after the onset of symptoms, as recommended by current guidelines (*1*,*15*).

Published studies report an enormous variability in how long after infection Zika virus RNA can be detected in semen, which makes advising patients on the risks for sexual transmission difficult. In this case, testing serial semen samples for evidence of Zika virus RNA helped guide patient management, and doing this routinely in returning travelers in non-Zika virus–endemic countries might be warranted. In such a context, a positive result should be interpreted as indicating possible ongoing risk for sexual transmission.

Further studies on the kinetics of virus isolation and Zika virus RNA detection from semen are needed to help inform guidelines on Zika virus sexual transmission and how to manage the risks. These studies would be especially useful in managing asymptomatic patients in whom evidence of Zika virus RNA detection, virus isolation, and sexual transmission is almost totally lacking.
